# The Effect of Beef Production System on the Health, Performance, Carcass Characteristics, and Meat Quality of Holstein Bulls

**DOI:** 10.3390/ani10101922

**Published:** 2020-10-19

**Authors:** Naomi H. Rutherford, Alan W. Gordon, Gareth Arnott, Francis O. Lively

**Affiliations:** 1 Agri-Food and Biosciences Institute, Large Park, Hillsborough, Co Down BT 26 6DR, Northern Ireland, UK; francis.lively@afbini.gov.uk; 2 Institute for Global Food Security, School of Biological Sciences, Queens University Belfast, Belfast BT9 5DL, Northern Ireland, UK; g.arnott@qub.ac.uk; 3 Agri-Food and Biosciences Institute, Newforge Lane, Belfast BT9 5PX, Northern Ireland, UK; alan.gordon@afbini.gov.uk

**Keywords:** concentrate, ad libitum, grazing, beef, finishing, cattle

## Abstract

**Simple Summary:**

Dairy-origin beef accounts for a considerable proportion of the prime beef supply. Yet, the beef production potential of dairy bred calves is declining following years of single trait selection for attributes such as milk yield. Producing these animals as bulls allows for increased growth rates and efficiency in comparison to steers, however these systems often involve a high level of concentrate feeding. Thus, creating a system that is subject to fluctuating feed prices and beef market volatility. This objective of this study was to evaluate four production systems, which differed in terms of diet during the grower period. The impacts on health, performance, carcass characteristics, and meat quality were all considered. Our research shows that ad libitum concentrate feeding throughout the production system resulted in superior carcass weights. Increasing the proportion of grazed grass in the diet during the grower period, did result in a reduced carcass weight in comparison to ad libitum concentrates, however total concentrate intake, and therefore the production costs during the grower period were also reduced. Production system had no effect on health or meat quality. In conclusion, including a gazing period within bull beef production systems may be a more sustainable approach to producing Holstein bulls.

**Abstract:**

The aim of this study was to evaluate the effect of production system on the health, performance, carcass characteristics, and meat quality of autumn born (AB) and spring born (SB) Holstein bulls. The study involved a total of 224 Holstein bulls and was conducted over two years (2017/18, 2018/19). The four production system treatments differed during the grower period and consisted of: (i) grazed with no concentrate supplementation (G), (ii) grazed with 2 kg concentrate supplementation per day (G2), (iii) grazed with ad libitum access to concentrates (GA) and (iv) housed with ad libitum access to concentrates and grass silage (HA). All bulls were finished on ad libitum concentrates and grass silage and were slaughtered at a mean age of 15.5 months. Total grower dry matter intake (DMI) (*p* < 0.001) and total finishing DMI (*p* < 0.001) differed between production systems for both AB and SB bulls, with that of GA bulls being the greatest in both cases. Average daily gain (ADG) during the grower period was greatest (*p* < 0.001) for the HA production system in the AB bulls and the GA and HA production systems for the SB bulls. However, during the finishing period, G bulls had the greatest (*p* < 0.001) ADG of the AB bulls, while that of the SB bulls was from the G2 production system (*p* < 0.001). For both AB and SB, bulls on the GA and HA production systems produced heavier cold carcass weights than the G and G2 bulls (*p* < 0.001). There was no significant difference (*p* > 0.05) in health, carcass conformation, fat classification, or meat quality between production systems.

## 1. Introduction

The UK dairy herd is predominantly of the Holstein-Friesian (HF) breed, with a major emphasis placed on maximising milk yield [1]. As a result of years of continued single-trait selection for milk yield, the beef production potential of HF dairy-bred calves has declined [2]. Yet, dairy-bred cattle account for a considerable proportion of the prime beef supply (cattle produced for the sole purpose of beef production) [3].

The ability for bulls to out-perform steers in terms of growth rate and feed efficiency has been widely documented within the literature. Steen [4] reported a 31% higher carcass gain for bulls, while improvements in feed conversion ratio (FCR) of 18.8% have been documented [5]. In addition, to meet UK market specifications bulls must be slaughtered under 16 months of age, therefore allowing for a quicker throughput of livestock on the farm. Although the utilisation of bulls allows for increased efficiency in beef production systems, they accounted for only 10% of the UK prime cattle slaughtered in 2017, with steers representing a considerable 53% [6]. One possible reason for the low proportion of male cattle being finished as bulls is that bull beef production is predominantly an intensive indoor system, involving a high level of concentrate feeding [7]. Feed costs have been shown to account for a substantial proportion of the variable costs in beef production with values of up to 75% being reported [8]. Consequently, these systems are subject to fluctuating concentrate prices and market volatility [9]. Thus, a production system must be aimed at reducing production costs, whilst maximising performance.

Grazed grass is the cheapest form of feed for ruminants [8], and thus, the inclusion of a grazing period during the first summer could help to reduce production costs. However, with bulls being characteristically leaner than steers [10], it is important that carcass weight and subcutaneous fat depth are not compromised. Thus, concentrate supplementation at pasture during the growing period may be required to ensure bulls meet market specification. In addition, the impact of production system on animal health should also be considered. Long term housing cattle on slatted flooring may have a negative impact on hoof health [11], while autumn housing of cattle after grazing can be a high risk period for pneumonia [12].

The objective of this study was to compare the health and performance of Holstein bulls on four differing production systems to identify if a grazing period could be included in Holstein bull beef production, and if concentrate supplementation at pasture was required. Specifically, we examine the hypothesis that performance will increase linearly with increasing concentrate supplementation at pasture, and that these differences in live weight will still be evident at slaughter. In addition, we hypothesise that pneumonia incidence will be greater at the beginning of the finishing period, for those bulls which have been grazed during the summer. Finally, due to a homogenous finishing period, meat quality will be unaffected by production system.

## 2. Materials and Methods

This study was undertaken from May 2017 until June 2019 at the Agri-Food and Biosciences Institute (AFBI), Hillsborough, Northern Ireland. All experimental procedures used in this study were conducted in compliance with the United Kingdom Animals (Scientific Procedures) Act 1986.

### 2.1. Experimental Design and Animal Management

This study involved of total of 224 Holstein bulls and was conducted over two years (2017/18 (*n* = 112) and 2018/19 (*n* = 112)). In each year a group of 56 autumn born (AB) and a group 56 spring born (SB) bull calves were selected. These bulls were assigned to one of four production system treatments, which differed during the grower period and had a homogenous finishing period, the durations of which are reported in Table 1. The four production system treatments included; (i) grazed with no concentrate supplementation (G), (ii) grazed with 2kg concentrate supplementation per day (G2), (iii) grazed with ad libitum access to concentrates (GA) and (iv) housed with ad libitum access to concentrates and grass silage (HA). Each treatment group consisted of 14 animals and was balanced for live weight (LW) and age. All bulls were housed and finished on a diet of ad libitum concentrates and grass silage before being slaughtered at a mean age of 15.5 months. Prior to the trial commencing, all animals were housed on straw bedding and managed similarly, with ad libitum access to grass silage and 2 kg per head per day (/h/d) concentrate supplementation from three weeks post-weaning until the beginning of the trial. The AB bulls commenced the trial approximately one month prior to the SB bulls, to allow SB bulls time to be weaned and adjust to a post-wean diet. Thus, AB bulls had a mean initial age of 192 ± 3.5 days at 212 ± 4.0 kg LW, while SB bulls were on average 116 ± 3.5 days of age at 119.6 ± 4.0 kg LW at the commencement of this trial.

### 2.2. Management during Grower Period

HA were housed in slatted accommodation, with access to cubicles. Grass silage was fed once daily and concentrates were fed in a trough. HA were initially offered 2 kg/h/d and gradually built up to ad libitum concentrate feeding, with concentrates being split into two feeds daily from 3 kg and 4 kg supplementation for the SB and AB groups, respectively until ad libitum at d28 for SB bulls and d56 for AB bulls. The SB bulls reached ad libitum concentrate intake earlier than AB bulls, as they were younger at the beginning of the trial and therefore initially had a lower dry matter intake (DMI).

G, G2, and GA were rotationally grazed in a 4 paddock system. A 7.2 ha mature perennial ryegrass pasture was divided into four equal blocks of 1.8 ha, with each block subdivided into a further six paddocks, three paddocks of 0.38 ha for AB bulls and three of 0.21 ha for SB bulls. Divisions between paddocks consisted of two strands of electric wire. Each of the six treatment groups at pasture were assigned one paddock per block. All cattle were grazed in one block at a time, and therefore were all rotated on the same day. Cattle were moved when one group (primarily G or G2) reached a post-grazing height of approximately 6.5 cm. A group of 30 spare cattle were then used to graze out the remaining paddocks (particularly GA) in order to bring all paddocks down to the same post-grazing cover. No live weight or intake data was recorded for the spare cattle.

Post turnout, G2 and GA were offered concentrates in a trough, G2 were fed 2 kg concentrates once daily throughout the trial. GA were gradually built up to ad libitum concentrate feeding, at the same rate and frequency as HA, at which point a creep feeder was introduced. The duration of the grower period is shown in Table 1, at the end of which G, G2, and G2 bulls were housed, and HA bulls were moved to the finishing house.

### 2.3. Management during Finishing Period

All bulls were housed on slatted accommodation during the finishing period, the mean duration of which is shown in Table 1. The dimensions of the pens were 3.4 m × 2.7 m. Each treatment group was split into three pens of four and one pen of two bulls and randomly assigned a pen number. All bulls were finished on ad libitum concentrates and grass silage. Therefore, GA and HA bulls continued on ad libitum concentrate feeding. The G and G2 bulls were initially offered 2 kg/h/d and 3 kg/h/d, respectively and gradually built up to ad libitum over a duration of seven and sex weeks, respectively. Concentrates were split into two equal feeds when supplementation was between 5 and 8 kg. Bulls were slaughtered at a mean age of 15.5 months.

### 2.4. Live Weight and Health

All bulls were weighed on two consecutive days prior to the commencement of this trial, at the end of the grower phase and at slaughter. The mean of the two weights was taken to be the animals’ LW on each of the three occasions. Throughout the trial bulls were weighed fortnightly to monitor performance. As two of the production system treatments included ad libitum concentrate feeding, bulls were always weighed full, at approximately the same time on each occasion. All weighbridges were calibrated to ISO9001 standards. Average daily gain (ADG) for each phase was determined by the difference between the initial LW and final LW, divided by duration (days) of each phase. Bulls were monitored daily for clinical signs of ill health. The incidence of pneumonia and lameness were recorded (each on a 2 point scale: 0 = no clinical signs and 1 = clinical signs observed) together with any antibiotic treatments given. Rumen temperature boluses (Thermobolus small, Medria, France) were administered at the beginning of this trial to 203 bulls. Temperature alerts from the boluses were used as an additional tool to assess health status.

### 2.5. Feed Intake and Composition

Pre- and post-grazing heights were measured using a Jenquip rising plate meter (model EC09) (Jenquip, New Zealand) and the difference used to estimate intake. Group intakes of silage and concentrates were recorded daily and refused feed was removed and weighed twice weekly prior to fresh feed being offered. A 10% fresh weight refusal rate was considered acceptable for ad libitum intakes. Mean estimated individual intakes were determined by dividing the group intake by the number of animals per group.

Pre- and post-grazing grass samples were analysed weekly using NIRS for crude protein (CP), acid detergent fibre (ADF), water soluble sugars (WSS), and metabolisable energy (ME) using the methods described by Park et al. [13] and Morrison et al. [14]. Daily silage and concentrate dry matter (DM) were monitored, together with that of the pre- and post-grazing grass samples. This was completed by placing samples in a forced-air circulation oven at 80 °C for 48 h. Dried concentrate and silage samples were bulked fortnightly to allow chemical composition (Ash, ADF, neutral detergent fibre (NDF) and gross energy) to be determined via a wet chemistry analysis. The concentrate used was a course beef blend with the main composition (g/kg) being maize 201, barley 199, distillers 198, corn gluten 149, soya hulls 47, sugar beet pulp 46, rapeseed 45, palm kernel 45.

### 2.6. Carcass Characteristics

Bulls were transported 42 km in a lorry to a commercial abattoir on the morning of slaughter. Bulls remained in one group during lairage and were then slaughtered using the commercial abattoir procedure [15]. At slaughter, carcass conformation and fat classification were recorded according the 15-point EU scoring system [16]. Carcass conformation was converted to a numerical scale, where 1 represents a P−, and 15 represents an E+ for conformation. For fat classification, 1 represented 1-, while 15 represented 5+. Internal fat (cod, kidney, and channel fat) was removed from the carcass and weighed during dressing [16], while carcass weight was recorded for each animal after dressing. Dressing percentage was calculated by dividing cold carcass weight by LW at slaughter. Carcass gain was calculated as follows:

$$\mathrm{Carcass}\;\mathrm{gain}=\frac{\mathrm{hot}\;\mathrm{carcass}\;\mathrm{weight}-\frac{\mathrm{initial}\;\mathrm{weight}}{\mathrm{dressing}\;\mathrm{percentage}}}{\mathrm{days}\;\mathrm{on}\;\mathrm{experiment}}$$

A post-mortem examination on the lungs, liver, and heart was conducted following slaughter by the veterinary inspector. Lungs were inspected for signs of pleurisy and pneumonia, the liver for abscesses and peritonitis, and the heart for pericarditis. Each organ was scored on a scale of 1 to 4 (1 = no signs, 2 = mild, 3 = moderate, and 4 = condemned).

At 24 h post-slaughter, carcasses were split between the 9th and 10th rib, and the *longissimus dorsi* muscle was scored for marbling using the Meat Standards Australia scoring system [17]. Subcutaneous fat depth (mm) was measured at three points (0.25, 0.50, and 0.75) along the length of the *longissimus dorsi* muscle on both sides of the carcass, the mean of these values was taken as the subcutaneous fat depth for each animal [1]. Carcass fat colour was also scored on each side of the carcass on a 5-point scale (1 = very white and 5 = very yellow) [18]; the mean of these values was taken as the carcass fat colour.

### 2.7. Instrumental Meat Quality

Two samples from the *longissimus dorsi* muscle were removed from the anterior fore-rib joint at three days post-slaughter [16]. These meat samples were aged at 3 °C, one until day 7 and the other until day 14 post-slaughter. At D7 ultimate pH (pH_ult_) and colour (L*(lightness), a*(redness) and b*(yellowness)) were assessed using a Jenway 370 pH meter and a Chroma Meter CR-400, respectively. Both instruments were calibrated prior to measurements being taken. The illuminant for the Chroma Meter was D_65_, while the angle of observation was 2°. D7 and D14 samples were then vacuum-packed and frozen, where they were stored until further measurements were taken.

Samples were left to thaw over a period of 24 h at 4 °C. Following this, they were removed from the packaging and left to bloom for 40 min. Samples were vacuum-packed and cooked in a water bath at 70 °C for 50 min. Samples were weighed pre- and post-cooking, and cooking loss was calculated as follows:

$$\mathrm{Cooking}\;\mathrm{loss}\;\%=\left(\frac{\mathrm{pre}\;\mathrm{cooking}\;\mathrm{weight}-\mathrm{post}\;\mathrm{cooking}\;\mathrm{weight}\;}{\mathrm{pre}\;\mathrm{cooking}\;\mathrm{weight}\;}\right)\;100$$

Warner Bratzler Shear Force (WBSF) measurements were completed on each sample using an Instron 3366 Universal Testing Instrument. Samples were cored parallel to the longitudinal orientation of the muscle fibres and ten cores (sub-samples) were taken from each meat sample. All sub-samples were of the same diameter (12.5 mm), and were sheared perpendicular to the muscle fibres. The mean maximum load of the 10 sub-samples was considered as the WBSF value for each meat sample.

### 2.8. Statistical Analysis

All statistical analysis was completed using Genstat (19th edition). Summary statistics were conducted on the composition of feed. The AB and SB bulls were analysed separately due to the differences in the durations of the grower and finishing periods outlined in Table 1, however, the methods and models of analysis were consistent for both AB and SB. Analysis of dry matter intakes was carried out using linear mixed model (LMM) methodology using the REML estimation method. Production system was fitted as a fixed effects, with year and day fitted as the random effects for grower intakes, while pen number was also included as random effect for the finishing intakes. A Fisher’s Least Significant Test was used to further assess pairwise differences between the individual levels of the effects. Live weight performance and FCR were also analysed using the REML estimation method. Fixed effects included initial age, initial weight and production system, while year was included as a random effect for the grower period, and year and pen number were used as random effects for parameters from the finishing period. Carcass characteristics and instrumental meat quality were analysed using the REML estimation method, with production system as a fixed effect and year and slaughter date as random effects. Again, in all cases multiple comparisons were conducted using a Fisher’s Least Significant Test.

Health variables (pneumonia incidence and lameness incidence) were modelled using generalised linear mixed model methodology with production system as a fixed effect. The random effects differed according to which period the health variables related to; for the grower period, year was used as a random, the finishing period consisted of year and pen number, while the slaughter health variables included year and slaughter date as random effects. The binary variables used a binomial distribution with a logit link function, while the count variables used a Poisson distribution with a logarithmic link function.

## 3. Results

### 3.1. Grower Period

The chemical composition of the feed offered throughout the study is shown in Table 2. DMI during the grower period followed similar trends for both AB and SB bulls (Table 3) with forage DMI being greatest for the G bulls (*p* < 0.001). The greatest concentrate DMI (*p* < 0.001) and total DMI (*p* < 0.001) were observed for the GA production system. The AB bulls saw the greatest ADG and housed weight for the HA production system (*p* < 0.001) (Table 4). For the SB bulls, GA and HA bulls had similar housed weights, while G SB bulls had the lightest (*p* < 0.001) housed weight and lowest ADG (*p* < 0.001) (Table 4).

### 3.2. Finishing Period

Forage, concentrate, and total DMI differed significantly (*p* < 0.001) between production systems during the finishing period, for both AB and SB bulls (Table 3). The AB bulls on the G production system displayed a superior ADG during the finishing period (*p* < 0.001), resulting in a slaughter live weight of 34.1 kg lighter than the GA bulls (*p* < 0.05). While the SB bulls saw a 68.5 kg difference between the G and GA bulls (*p* < 0.001).

### 3.3. Overall DMI and Performance

Total concentrate DMI (Table 3) shows that sizeable reductions (*p* < 0.001) in concentrate usage can be achieved of up to 650 kg for AB bulls and up to 890 kg for SB bulls between the G and GA production systems. Table 4 shows that even when the grower and finishing periods are combined differences in overall ADG were observed between production systems for both AB (*p* < 0.05) and SB bulls (*p* < 0.001). In the case of the SB production systems, the lowest ADG was observed from the G production system, while that of GA and HA were the greatest.

### 3.4. Carcass Characteristics

For the AB bulls those on the GA and HA production system had a greater (*p* < 0.001) carcass hot weight and cold weight than G and G2 (Table 5). Carcass gain also differed (*p* < 0.05) between production systems for AB bulls, together with the weight of internal fat (*p* < 0.001). The remaining carcass traits and meat quality parameters were not significantly different (*p* > 0.05).

Similar results were obtained for the SB bulls, with the cold carcass weight of the G bulls being significantly (*p* < 0.001) lighter than the other three production systems (*p* < 0.001). Carcass gain for this production system also proved to be the lowest of the four SB production systems. Again there was no significant difference (*p* > 0.05) between the remaining carcass traits or meat quality parameters.

### 3.5. Health

The results for the health variables are not presented as there was no significant difference (*p* > 0.05) in the incidence of pneumonia or lameness during either the grower or finisher period for both AB and SB bulls. Furthermore, a number of health variables from the grower and finisher period were so insignificant that they could not be analysed. In addition, no significant (*p* > 0.05) difference in the incidence of liver abscesses, pericarditis, or pneumonia were identified during a post-mortem examination of AB and SB bulls.

## 4. Discussion

### 4.1. Grower Period

Pasture based intakes for growing dairy bred bulls are not widely reported within the literature. Holstein-Friesian heifers on a grass only diet have been reported to have a pasture DMI of 5.37 ± 0.394 kg/d from the age of 5–10 months [14]. These heifers were of a similar age to the AB bulls in the current study, yet displayed a greater forage DMI than those on the G production system. However, it should be noted that different methods for calculating grazed grass intake exist between the two studies. Furthermore, pasture based intakes in the current study were based on a group estimate, which could be considered a limitation. However, the accurate recording of individual intakes at pasture remains a challenge under field conditions [19].

For both AB and SB bulls the G production system had the greatest forage intake. Supplementing concentrates is known to result in the substitution of forage intakes, with substitution rates increasing as the amount of concentrates offered increases [20]. Thus, the lower forage intakes of the G2, GA and HA production systems is to be expected. Bulls on the G production system, did however have the lowest total DMI during the grower period. Forage based diets are known to result in a lower total DMI in comparison to that of an ad libitum concentrate diet [21,22]. This is primarily due to the bulky fibrous nature of the forage feedstuff which slows the rate of passage through the rumen and therefore restricts intakes [5,23].

The level of performance during the grower period is consistent with previous research. Keane and Fallon [24] observed an ADG of 0.72 kg/d during an unsupplemented 203 day grazing period. Similarly, during their first summer SB Holstein-Friesian steers achieved an ADG of 0.68kg/d on a grass only diet [25]. Murphy et al. [26] reported an ADG of 0.88 kg/d for SB bulls offered 2 kg concentrate supplementation at pasture, which is similar to that observed from SB G2 bulls in the current study. The HA production system resulted in an ADG of more than double that of G for both the AB and SB bulls. This clearly demonstrates the growth potential of Holstein bull calves when offered a nutrient dense diet. Similar levels of performance from ad libitum concentrate feeding from 3 to 8 months of age were achieved by Therkildsen et al. [27]. While that reported by Keane and Fallon [24] was slightly lower (1.32 kg/d) than the level of performance achieved in the current study. The greater ADG of AB HA in comparison to AB GA may be due HA bulls being housed and therefore having considerably less energy expenditure due to exercise [28]. Furthermore, they were not exposed to the unfavourable weather conditions that the other treatments were occasionally subjected to [29].

### 4.2. Finishing Period

Although variation in intakes occurred between production systems for both AB and SB bulls, as a whole they were consistent with previous research [24,28,30]. The ADG AB bulls on the HA production system during the finishing period, is somewhat lower that that achieved during the grower period. This is consistent with other studies where growth rates do not remain consistently high throughout the full production system [24,31]. The sigmoidal growth curve of cattle illustrates that growth rates reduce as an animal approach maturity [32], with increased maintenance requirements and fat deposition being contributing factors [1]. Although these bulls had not yet reached full mature weight, the expression of this trait is thought to be more evident when the diet remains consistent throughout the production system [33].

In contrast, the G and G2 bulls (for both AB and SB) saw a considerable increase in ADG during the finishing period. This was somewhat greater than that achieved by the HA bulls, and that which has previously been reported within the literature [24,34]. These results would imply that the G and G2 bulls exhibited some level of compensatory growth during the finishing period [31,35,36]. However, overall DWLG and slaughter weights were still lower for G and G2 bulls, thus indicating that these bulls did not fully compensate during the finishing period. This is in agreement with previous research, which indicates that a longer finishing period may be required for bulls to reach the same LW as those on the GA and HA production system [31,36]. However, the nature of the production systems used in this study, would lead to differences in slaughter weight being expected, which could be offset against the differences in total concentrate intake, and thus production costs. 

Kirkland et al. [5] reported an overall ADG of 1.33 ± 0.043 kg/d for bulls slaughtered at 550 kg LW and offered ad libitum concentrates and barley straw throughout the study. These bulls were of a similar age (112 ± 26.0 days) at the beginning of the trial to the SB bulls in the current study. The overall ADG of the GA and HA SB bulls are consistent with that of Kirkland et al. [5]. That of the G, G2, and HA AB bulls are also comparable, indicating that similar levels of overall performance can be achieved from a range of production systems.

### 4.3. Carcass Characteristics and Meat Quality

The SB bulls on G and G2 production systems reached similar slaughter and carcass weights as those slaughtered at 16 months on a 50:50 silage to concentrate diet by Kirkland et al. [1]. While the slaughter and carcass weights of GA and HA SB bulls are consistent with that of the ad libitum fed bulls of Manni et al. [31].

Although there was no difference in carcass fat classification or subcutaneous fat depth, differences in internal fat were observed for AB bulls. This contrasts with the results of Therkildsen et al. [27] who found all three fat measurements to be significantly reduced for Friesian bulls that had spent a season at pasture. However, the longer finishing period in the current study, could be expected to diminish the effects of the grower period. Furthermore, Kirkland et al. [5] highlighted that differences in fat deposition location could be as a result of breed. With Holstein cattle having a greater tendency for the deposition of fat in internal depots rather than subcutaneous depots, compared to Friesian, or continental bred cattle [2,5]. The variation in internal fat weight for AB bulls, is likely explained by the differences in slaughter weight. This linear relationship has previously been reported in the literature [5,37]. Fat colour did not differ according to production system. Previous research has shown that slaughtering bulls off pasture results in a yellower fat colour than that of bulls intensively finished [27]. However an intensive finishing period of 10 weeks is sufficient to counteract any differences [27]. Furthermore, production system had no effect on meat quality, and therefore if any differences were apparent after the growing period, these were eliminated during the finishing period.

### 4.4. Health

Health status and pneumonia incidence were not impacted by production system in this study which is in agreement with previous research [31]. Furthermore, the non-significant incidence of liver abscesses between production systems indicates that the level of concentrate feeding did not have any adverse effects. Bulls were gradually built up to ad libitum concentrates in this study, thus allowing the rumen time to adjust. This, together with the provision of adequate forage in the diet, is thought to be key in preventing nutritional disorders of this kind [38].

## 5. Conclusions

In conclusion, the results of this study demonstrate that both AB and SB bulls on ad libitum concentrates (GA and HA) had superior performance during the grower period achieving ADGs of up to 1.58 kg/d. In contrast, AB G and SB G2 bulls had elevated growth rates during the finishing period (1.48 kg/d and 1.43 kg/d, respectively), and likely exhibited a degree of compensatory growth during this time. However, differences in cold carcass weight were still revealed, with that of G and G2 being lighter than GA and HA, for both AB and SB bulls. Nevertheless, G and G2 production systems did result in a lower total concentrate intake than GA and HA, which would assist in reducing production costs. Instrumental meat quality and animal health were unaffected in this study.

## Figures and Tables

**Table 1:** Mean durations (±SD) in days of each period for autumn born (AB) and spring born (SB) bulls.

Season	AB	SB
Grower	89 ± 1.0	149 ± 10.5
Finisher	191 ± 25.6	209 ± 31.7

AB = autumn born; SB = spring born.

**Table 2:** Mean chemical composition of feed offered to bulls.

Chemical Composition	Concentrates	Grass	Grass Silage
DM (g/kg)	873.6	257.8	282.5
Ash (g/kg DM)	77.41		110.6
Gross energy (MJ/kg DM)	18.31		18.74
Nitrogen (g/kg DM)	28.84		22.66
ADF (g/kg DM)	134.1		333.1
NDF (g/kg DM)	317.5		563.4
CP (%DM)		15.84	
ADF (%DM)		32.12	
WSS (%DM)		12.92	
ME (MJ/kgDM)		10.74	

DM = dry matter; ADF = acid detergent fibre; NDF = neutral detergent fibre; CP = crude protein; WSS = water soluble sugars; ME = metabolisable energy.

**Table 3:** Mean estimated dry matter intakes during the grower and finishing period for autumn and spring born bulls on four production systems.

Production System		Autumn Born				SED	Spring Born				SED	*p*-Value	
		G	G2	GA	HA		G	G2	GA	HA		AB	SB
Grower DMI (kg/d)	Forage	3.49 ^c^	3.15 ^c^	2.24 ^b^	1.58 ^a^	0.145	3.08 ^c^	2.97 ^c^	1.53 ^b^	0.83 ^a^	0.116	<0.001	<0.001
	Concentrate	-	1.63 ^a^	6.33 ^c^	5.71 ^b^	0.212	-	1.68 ^a^	5.35 ^c^	5.05 ^b^	0.122	<0.001	<0.001
	Total	3.49 ^a^	4.78 ^b^	8.57 ^d^	7.29 ^c^	0.231	3.06 ^a^	4.65 ^b^	6.84 ^d^	5.87 ^c^	0.138	<0.001	<0.001
Finishing DMI (kg/d)	Forage	1.97 ^b^	1.66 ^a^	1.75 ^a^	1.74 ^a^	0.077	1.67 ^a^	2.25 ^b^	2.07 ^b^	2.48 ^c^	0.091	<0.001	<0.001
	Concentrate	8.29 ^a^	8.04 ^a^	9.51 ^b^	8.06 ^a^	0.151	6.41 ^a^	7.28 ^c^	7.92 ^d^	6.98 ^b^	0.152	<0.001	<0.001
	Total	10.17 ^b^	9.58 ^a^	11.28 ^c^	9.70 ^a^	0.167	7.85 ^a^	9.38 ^b^	10.03 ^c^	9.35 ^b^	0.178	<0.001	<0.001
Total concentrate DMI (t)		1.70 ^a^	1.79 ^a^	2.35 ^b^	2.24 ^b^	0.117	1.44 ^a^	1.84 ^b^	2.33 ^c^	2.20 ^c^	0.067	<0.001	<0.001

G = Grazed; G2 = Grazed +2kg; GA = Grazed + ad libitum concentrates; HA = Housed + ad libitum concentrates; AB = autumn born; SB = spring born; DMI = dry matter intake. Superscripts ^a–d^ represent significant differences between production systems for either AB or SB bulls at the *p*-value shown.

**Table 4:** Mean performance of autumn and spring born bulls on four production systems.

Production System		Autumn Born				SED	Spring Born				SED	*p*-Value	
		G	G2	GA	HA		G	G2	GA	HA		AB	SB
Live weight (kg)	Initial	209.8	210.2	213.4	212.4	8.92	119.2	120.4	118.9	119.7	6.90	ns	ns
	Housed	275.3 ^a^	299.8 ^b^	333.8 ^c^	351.8 ^d^	5.76	217.5 ^a^	248.5 ^b^	308.3 ^c^	318.1 ^c^	6.90	<0.001	<0.001
	Slaughter	580.6 ^a^	575.0 ^a^	614.7 ^b^	599.6 ^ab^	13.82	510.3 ^a^	548.1 ^b^	578.8 ^c^	578.6 ^c^	13.38	<0.05	<0.001
ADG (kg/d)	Grower	0.72 ^a^	0.99 ^b^	1.38 ^c^	1.58 ^d^	0.065	0.66 ^a^	0.87 ^b^	1.27 ^c^	1.34 ^c^	0.047	<0.001	<0.001
	Finishing	1.61 ^c^	1.48 ^b^	1.46 ^b^	1.29 ^a^	0.060	1.38 ^bc^	1.43 ^c^	1.28 ^ab^	1.22 ^a^	0.051	<0.001	<0.001
	Overall	1.32 ^a^	1.31 ^a^	1.44 ^b^	1.39 ^ab^	0.050	1.08 ^a^	1.20 ^b^	1.28 ^c^	1.28 ^c^	0.037	<0.05	<0.001

G = Grazed; G2 = Grazed +2kg; GA = Grazed + ad libitum concentrates; HA = Housed + ad libitum concentrates; AB = autumn born; SB = spring born; ADG = average daily gain. Grower: Initial to housing; Finishing: Housing to slaughter; Overall: Initial to slaughter. Superscripts ^a–d^ represent significant differences between production systems for either AB or SB bulls at the *p*-value shown.

**Table 5:** Mean carcass characteristics and meat quality of autumn and spring born bulls on four production systems.

Production System	Autumn Born				SED	Spring Born				SED	*p*-Value	
	G	G2	GA	HA		G	G2	GA	HA		AB	SB
Age at slaughter (d)	472.4	470.4	473.1	471.4	1.75	476.4	472.6	473.4	472.9	3.11	ns	ns
Hot carcass weight (kg)	297.2 ^a^	300.7 ^a^	322.1 ^b^	319.6 ^b^	7.64	263.5 ^a^	287.1 ^b^	301.5 ^b^	302.4 ^b^	7.89	<0.001	<0.001
Cold carcass weight (kg)	291.3 ^a^	294.7 ^a^	314.5 ^b^	313.3 ^b^	7.60	258.2 ^a^	281.4 ^a^	295.5 ^b^	296.4 ^b^	7.73	<0.001	<0.001
Carcass gain (kg/d)	0.69 ^a^	0.69 ^a^	0.76 ^b^	0.74 ^ab^	0.027	0.56 ^a^	0.63 ^b^	0.67 ^bc^	0.68 ^c^	0.212	<0.05	<0.001
Carcass conformation classification ^1^	4.35	4.04	4.31	4.42	0.209	3.77	3.89	4.12	4.19	0.270	ns	ns
Carcass fat classification ^2^	7.74	7.52	8.09	7.24	0.429	6.44	7.00	6.86	6.77	0.378	ns	ns
Dressing proportion (g/kg)	504.0	506.9	510.3	513.8	4.22	503.1	508.5	506.3	511.9	4.96	ns	ns
Internal fat (kg)	19.08 ^ab^	18.02 ^a^	22.43 ^c^	21.11 ^bc^	1.120	16.97	17.53	18.92	18.05	1.220	<0.001	ns
Subcutaneous fat depth (mm)	7.38	7.49	8.21	8.15	0.669	6.85	6.59	7.40	6.64	0.495	ns	ns
MSA marbling score	419.9	418.5	458.8	448.1	32.26	401.4	428.3	429.6	416.3	34.4	ns	ns
Carcass fat colour ^3^	2.42	2.55	2.59	2.48	0.123	2.35	2.56	2.47	2.69	0.149	ns	ns
Ultimate pH	5.63	5.75	5.64	5.69	0.063	5.73	5.82	5.73	5.90	0.094	ns	ns
Colour-L*	39.57	37.94	39.20	39.03	0.830	38.94	38.50	38.15	38.06	0.848	ns	ns
Colour-a*	27.26	26.12	26.97	26.18	0.575	25.99	26.05	25.89	25.12	0.677	ns	ns
Colour-b*	10.93	10.00	10.80	10.25	0.477	9.90	9.86	9.79	9.46	0.519	ns	ns
Cooking loss D7 (%)	27.20	25.25	26.27	25.47	0.877	25.09	25.07	24.84	24.09	0.949	ns	ns
Cooking loss D14 (%)	27.58	26.31	27.50	26.39	0.665	25.97	25.60	25.81	25.01	1.149	ns	ns
WBSF D7 (kgF)	4.70	4.50	4.65	4.87	0.283	4.83	4.67	4.36	4.52	0.313	ns	ns
WBSF D14 (kgF)	4.19	4.29	4.15	4.46	0.207	4.40	4.26	4.12	4.23	0.256	ns	ns

G = Grazed; G2 = Grazed +2kg; GA = Grazed + ad libitum concentrates; HA = Housed + ad libitum concentrates; AB = autumn born; SB = spring born; MSA = Meat Standards Australia; D7 = Day 7; D14 = Day 14; WBSF = Warner Bratzler Shear Force. ^1^ Score 1–15 (1 = P-, 15 = E+); ^2^ Score 1–15 (1 = 1-, 15 = 5+); ^3^ Score 1–5 (1 = pale, 5 = dark). Superscripts ^a–c^ represent significant differences between production systems for either AB or SB bulls at the *p*-value shown.
